# Next Generation Sequencing and Machine Learning Technologies Are Painting the Epigenetic Portrait of Glioblastoma

**DOI:** 10.3389/fonc.2020.00798

**Published:** 2020-05-15

**Authors:** Ivana Jovčevska

**Affiliations:** Medical Centre for Molecular Biology, Institute of Biochemistry, Faculty of Medicine, University of Ljubljana, Ljubljana, Slovenia

**Keywords:** glioblastoma, next generation sequencing, diagnosis, therapy, methylation, epigenetics, machine learning, deep learning

## Abstract

Even with a rare occurrence of only 1.35% of cancer cases in the United States of America, brain tumors are considered as one of the most lethal malignancies. The most aggressive and invasive type of brain tumor, glioblastoma, accounts for 60–70% of all gliomas and presents with life expectancy of only 12–18 months. Despite trimodal treatment and advances in diagnostic and therapeutic methods, there are no significant changes in patient outcome. Our understanding of glioblastoma was significantly improved with the introduction of next generation sequencing technologies. This led to the identification of different genetic and molecular subtypes, which greatly improve glioblastoma diagnosis. Still, because of the poor life expectancy, novel diagnostic, and treatment methods are broadly explored. Epigenetic modifications like methylation and changes in histone acetylation are such examples. Recently, in addition to genetic and molecular characteristics, epigenetic profiling of glioblastomas is also used for sample classification. Further advancement of next generation sequencing technologies is expected to identify in detail the epigenetic signature of glioblastoma that can open up new therapeutic opportunities for glioblastoma patients. This should be complemented with the use of computational power i.e., machine and deep learning algorithms for objective diagnostics and design of individualized therapies. Using a combination of phenotypic, genotypic, and epigenetic parameters in glioblastoma diagnostics will bring us closer to precision medicine where therapies will be tailored to suit the genetic profile and epigenetic signature of the tumor, which will grant longer life expectancy and better quality of life. Still, a number of obstacles including potential bias, availability of data for minorities in heterogeneous populations, data protection, and validation and independent testing of the learning algorithms have to be overcome on the way.

## Introduction

This review starts with outlining the complex nature of glioblastoma by providing brief information about its occurrence, mortality rate, molecular features, and heterogeneity. Our level of understanding of glioblastoma genetics has remarkably increased since the introduction of next generation sequencing. However, lack of effective patient treatment necessitates implementation of modern diagnostic and treatment options using newer technological developments. One such example is exploration of epigenetic markers for glioblastoma diagnosis and treatment. Although epigenetics in glioblastoma is still at its infancy, it shows potential for development of novel therapies. Moreover, development of machine learning and deep learning algorithms for glioblastoma patient care may improve objective disease diagnosis and can contribute to tailoring the most effective treatment based on patient molecular profile i.e., precision medicine. At last, understanding the molecular background of each patient will raise the quality of clinical care from the current supportive classical treatment to the level of significantly improving patient life expectancy and quality of life.

### Glioblastoma

In the United States, cancer is the second leading cause of death in both genders with the four most prevalent types being lung, breast, prostate, and colorectal, while brain cancers account for only about 1.35% of the cases (1, 2). However, contrary to their rare occurrence, in the year 2016 brain tumors were the major cause of cancer-related death among men younger than 40 years of age and women younger than 20 years of age (2). With 26% and 21% of the cases, brain and other nervous system tumors represent one of the most commonly diagnosed tumors in children and adolescents, respectively (2). Among adults, gliomas account for almost 80% of the primary malignant brain tumors (3). Gliomas can be classified based on location, differentiation pattern, anaplasia, mitotic activity, and necrosis. Moreover, according to the World Health Organization (WHO), histologically they progress from benign (WHO grade I and II) to malignant (WHO grade III and IV) (3). The most malignant type is the grade IV glioblastoma which accounts for 60–70% of all gliomas (4) and 16% of all primary brain tumors (5). The age-adjusted annual incidence of glioblastoma is 3.19 per 100,000 people in the United States (6). Glioblastoma is most commonly diagnosed in elderly Caucasian men with mean age of 64 years (6–8). Genetically there are two main glioblastoma subtypes—isocitrate dehydrogenase (IDH) wild type (or primary) and mutant (or secondary) that are histo-pathologically the same, but with different clinical progression (9, 10). *IDH*-mutant glioblastomas tend to develop from previously diagnosed WHO grade II or III gliomas and appear in patients younger than 50 years of age, while *IDH*-wild type glioblastomas appear *de novo* in patients with median age of 60 years (3, 11). In general, patients with *IDH*-mutant glioblastomas show better overall survival than patients with *IDH*-wild type tumors (11).

### Glioblastoma Diagnosis and Therapy

Due to unspecific symptoms like numbness, mood swings, fatigue, and mild memory loss (12), glioblastoma is usually diagnosed at an advanced stage, when little can be done for the patient. Definitive diagnosis can only be done histologically, but needs to be complemented with the recent advances in the molecular classification. The latest WHO classification of brain tumors (13) constitutes a combination of phenotypic and molecular parameters of brain tumors, and that leads to greater diagnostic accuracy. Still, in cases where molecular diagnostic testing is not available or is inconclusive, brain tumors are labeled as “not otherwise specified (NOS).” Standard treatment comprises maximal surgical resection, followed by concomitant chemotherapy with temozolomide and radiation, and then adjuvant chemotherapy (12). Temozolomide was approved by the US Food and Drug Administration (FDA) for treatment of glioblastoma in the year 2005 (14). It is a small lipophilic molecule that is absorbed completely after oral administration, minimally binds to plasma proteins and is able to penetrate the blood brain barrier (15). Still, this aggressive treatment gives patients only 12–18 months *post* diagnosis (16, 17), while the 5-year survival is only 9.8% (17). High mortality rate is a result of the localization and rapid tumor growth (3). In order to improve patient care and life expectancy, numerous alternative treatments such as tumor treating fields (18–20), gamma knife radiosurgery (21), and immunotherapy (22–25) are currently being explored.

## DNA Sequencing

### Sanger Sequencing

The first commercialized method for DNA sequencing named Sanger sequencing (26) was extensively used for almost three decades. Sanger sequencing or chain-termination sequencing is based on the use of 2′-deoxynucleotides (dNTPs) and 2′,3′-dideoxynucleotides (ddNTPs) for synthesis and termination of synthesis of the complementary DNA template, respectively. This leads to generation of fragments with different sizes which are separated by high-resolution gel electrophoresis and analyzed to reveal the DNA sequence. Automated Sanger sequencing used fluorescently labeled primers or terminating ddNTPs. Excitation of the fluorophores produced fluorescent emission in different colors that that were used for revealing the DNA sequence. One of the greatest accomplishments of automated Sanger sequencing was sequencing the complete human genome (27) that is considered the largest project of today's mankind (28). Still, its limitations in terms of cost, time, low throughput, efficiency and sensitivity, drove the development of newer sequencing technologies collectively named “next generation sequencing” (NGS).

### “Next Generation Sequencing” Boom

NGS methods are based on the same principle as Sanger sequencing: they both use polymerases for synthesis, modified nucleotides, and fluorescent detection (29). However, for some NGS platforms like Illumina, Life Technologies SOLiD, Ion Torrent Personal Genome Machine (PGM), and Roche 454 systems, the DNA template has to be clonally amplified prior to sequencing, while for the more sensitive Heliscope and Pacific Biosciences (PacBio) single molecule real-time (SMRT) systems pre-amplification is not needed (30). Different NGS platforms use different chemistry for library preparation and sequencing (31). For example, Illumina sequencers are based on the “sequencing by synthesis” approach with fluorescently labeled reversible nucleotide terminator chemistry (32). On the other hand, Ion torrent technology generates sequence templates on a bead or sphere via emulsion PCR with sequencing-by-ligation approach and proton release detection. At last, PacBio sequencers are based on SMRT sequencing with fluorescent detection (30). One of the major advantages of NGS is increased throughput at decreased expenditure i.e., its ability to generate large amount of data at reasonable costs. As an example, the standard Sanger sequencing yielded ~6 Mb DNA sequence per day at a cost of $500/1 Mb while NGS sequencers like Illumina GA (San Diego, CA, USA), yield ~5,000 Mb DNA sequence per day at a cost of $0.50/1 Mb (33). Still, potential problems that arise are setting the necessary infrastructure for NGS including machinery, costs for reagents, space for sample processing, and data storage (34). Moreover, trained personnel with adequate understanding of the software for data analysis and interpretation is a necessity. A more complex problem that should not be neglected is who owns the genetic information obtained from such analysis, and to what extent the raw data can be used for other pathologies besides the one originally intended? These limitations of NGS are issues that still need to be addressed together with institutional ethics boards, researchers and participants as the technology develops.

How did NGS become so popular? After the introduction of commercial next generation sequencers in the year 2005 (29), a new age in nucleic acid research was started. NGS is suitable for a wide range of applications in particular for analysis of genetic variations and mutations, mRNA, non-coding RNA, methylation studies as well as chromatin immunoprecipitation (ChIP)-sequencing (35). By generating genetic information from different individuals, NGS made it possible to perform large scale, comparative, and evolutionary studies (36), and also helped in the development of pharmaceuticals (37). Moreover, NGS started the era of genomic medicine—incorporating patient's tumor genetic information for diagnosis, treatment, and prevention of diseases. NGS can help in overcoming treatment challenges by identifying druggable genetic targets. At last, with NGS the human genome can be resequenced in order to get deeper understanding of how genetic changes affect health and disease (38). NGS offers enormous possibilities for increasing our understanding of human genetics of health and disease, which will change the way we diagnose disease and treat individuals.

### Third Generation Sequencing

The best method for identification of genetic variations crucial for disease development is DNA sequencing. The right sequencing method is desired to be high-throughput, low-cost and able to sequence long reads with high accuracy (39). Despite all the advantages that next generation sequencing offers, the short length of the obtained reads is its weak spot. This led to the emergence of a third generation of sequencing that enables single molecule long reads (39) such as SMRT sequencing by PacBio (40) and nanopore sequencing originally introduced in the year 1996 (41). The authors showed that with an electrical field, single-stranded DNA (ssDNA), or RNA molecules can be driven through a 1–10 nm ion channel, i.e., nanopore, in a lipid bilayer or membrane. When passing through the nanopore, different bases of the DNA strand will cause specific fluctuations in the electrical current; these signals can be converted to DNA sequence information (39). The advantage of this method is the short time for sample preparation, electrical, or fluorescent readout and reads in length of several kilobases of single DNA molecules in real time (29). However, there are two possible issues that have to be resolved: (1) Length of the recognition region of the nanopore should not be larger than 0.5 nm—size that is equivalent to the base-spacing in a ssDNA, otherwise signal interference from adjacent bases will be observed (39); and (2) The current speed of DNA translocation, 300 bases/ms, is too fast for individual bases to be identified—ideally it should be adjusted to 1 base/ms (39). A solution has been proposed by IBM (New York, NY, USA) by creating a nanopore matrix i.e., a transistor with alternating fields of metal and dielectric materials which can control the speed of DNA translocation (42, 43).

New technological developments in the sequencing fields offer different techniques for establishing patients' tumors' molecular profiles which are expected to accelerate the development of personalized medicine. For example, targeted sequencing will allow detection of specific genetic changes of a predefined set of genes; whole exome sequencing will provide information about the coding regions of genes of interest; while RNA sequencing will give information about the post-transcriptional changes (44). At last, whole genome sequencing will provide a complete in depth genetic picture for each patient, but at the cost of great computational power, time, and resources. Because of the high molecular diversity of glioblastoma, these technological advancements are expected to deepen our knowledge of its mechanisms of development and progression. At last, by understanding how the disease works at different molecular levels (transcriptomic, genetic, epigenetic, and protein), new more powerful drugs can be designed that will be of a great benefit for the patients.

## Paving the Road to Precision Medicine

Cancer is a complex disease which arises as a result of combination of hereditary i.e., genetic and environmental factors such as physical and chemical agents, diet, lifestyle, tobacco, and alcohol use (35). These interactions leave footprints in the genome either as mutations or as epigenetic modifications. Genetic changes range from single base substitutions to major chromosomal losses, while the epigenetic modifications influence gene expression as well as DNA replication and repair (45, 46).

Glioblastoma is a disease that is characterized with heterogeneity at both intra- and inter-tumoral level. As a response to its complexity, the scientific society moves away from identification of a single gene as a cause of a disease, and toward identification of combination of molecular changes that eventually lead to tumor development (35). Such changes can be commonly observed in different individuals with the same disease. Personalized medicine implies development of drugs for the needs of a single patient, while precision medicine refers to the classification (or diagnosing) of individuals into genomic subclasses which can be treated in more targeted i.e., precise ways (47). The advantage of using NGS for diagnostics is the simultaneous detection of a number of different markers, which otherwise requires separate consecutive tests and prolongs the diagnostic process.

### Genetics of Glioblastoma

During the course of The Cancer Genome Atlas (TCGA) project glioblastomas were genetically characterized. The most important findings included changes in three core pathways receptor tyrosine kinase (RTK)/rat sarcoma (RAS)/PI3K, p53, and retinoblastoma (RB) with alterations in 88, 78, and 87% of the analyzed cases, respectively (48). The most frequent gene alterations were found in epidermal growth factor receptor (*EGFR*—mutation in 45% of the cases), phosphatase and tensin homolog (*PTEN*—inactivated in 36% of the cases), cyclin-dependent kinase inhibitor 2A (*CDKN2A*—inactivated in 52% of the cases), cyclin-dependent kinase inhibitor 2B (*CDKN2B*—inactivated in 52% of the cases), tumor protein p53 (*TP53*—mutated in 35% of the cases) and *RB* (homozygote deletion in 11% of the cases) (11, 48). *IDH* mutations are rare in primary glioblastoma patients with *EGFR* and *PTEN* alterations, but are commonly found in low grade gliomas and together with *TP53* mutations in high grade gliomas that evolved from low grade gliomas (49). Later, Verhaak et al. established the molecular profile of glioblastoma (50). In their study, using molecular profiling they defined four glioblastoma subtypes: classical, proneural, neural, and mesenchymal with different molecular properties. This illustrates high glioblastoma heterogeneity at the molecular level that is present both within (intra) and among (inter) tumors.

Examining intratumor heterogeneity can be precisely performed with method that allows for analysis at individual cell resolution level, such as single-cell RNA sequencing (scRNA-seq) (51). scRNA-seq can be used not only to analyze tumor cells, but also non-tumor cells that shape the microenvironment and aid in tumor progression (52, 53). Numerous research groups are already using this technology to “shred glioblastomas to single-cells” and contribute to their molecular understanding (51, 54–58). One such study identified presence of different cells within the tumor compared to cells from the surrounding based on macrophage and microglia gene expression profiles (52). In another study (59) the authors provided evidence that glioblastoma stem cells shape the transcriptional and cellular landscapes of the tumor. In a different study (51), the authors proposed potential role of the expression levels of rare genes in glioblastoma tumorigenesis. Using the knowledge about molecular heterogeneity of glioblastoma, institutes already implemented next generation sequencing panels containing a specific set of glioblastoma-specific genes for patient diagnosis (60). It is reasoned that the intratumoral heterogeneity reflects the existence of different cellular subclones within the same tumor—this is why deciding the therapeutic strategy from a single biopsy specimen may not be enough for successful therapy (61). Moreover, the molecular diversity of glioblastoma is further increased during treatment—namely, tumors of patients treated with temozolomide present with a hypermutation phenotype (62, 63) which is associated with promotion of tumor growth and metastasis (64). At last, transcriptome analyses are also used for defining glioblastoma signatures that will help in precise disease diagnosis, as well as to anticipate therapy response and patient outcome (53).

### Epigenetics of Glioblastoma

Epigenetic modifications are heritable changes that affect gene expression, but do not change the DNA sequence itself (65, 66). Such changes are DNA methylation, histone modifications, and chromatin remodeling (67). Histones are positively charged proteins H1, H2A, H2B, H3, and H4 (68). Chromatin refers to the complex of negatively charged DNA and positively charged histone proteins, or the fundamental subunit “nucleosome” in the nucleus. Every nucleosome consists of about 146–147 bp DNA associated with octameric core of histone proteins—two H3-H4 histone dimers surrounded by two H2A-H2B dimers (69). Histone acetylation i.e., addition of acetyl groups to lysines of H3 or H4, weakens the interactions between histones and DNA which opens the accessibility to the transcription apparatus, while histone deacetylation removes the acetyl groups which results in histone condensation and gene inactivation (70). These dynamic processes are maintained by histone acetyltransferases (HAT) and deacetylases (HDAC). Histone modifications are different in pediatric and adult gliomas. In pediatric gliomas, the most common mutations are K27M and G34R/V on histone variant H3.3 (71). Mutations in *H3F3A* show specificity for pediatric high grade gliomas, and are not found in pediatric low grade gliomas, embryonic tumors, or ependymomas nor in adult glioblastoma (72). In adult gliomas, *IDH1* mutations indirectly affect H3K27 or H3K36 methylation (73). Lysine (K) methylation at positions K4, K36, and K39 on H3 marks active chromatin regions, while at positions K9 and K27 at H3 it marks inactive chromatin regions (74, 75); still, lysine methylation does not change the net charge of the histone tail (76). Another epigenetic modification is chromatin remodeling that causes conformation changes in chromatin which regulate the DNA-dependent processes, replication, and repair systems as well as centromere and telomere maintenance (67, 77). These 3D conformational chromatin changes can affect gene expression by regulating access to RNA polymerases and transcription factors (77). Examples of the involvement of chromatin remodeling in glioblastoma pathogenesis are switch/sucrose non-fermenting (SWI/SNF) core complex (78) and Brahma-related gene 1 (BRG1), one of the catalytic subunits of the SWI/SNF chromatin remodeling complex (79) that regulate stemness and tumorigenic potential of glioma initiating cells.

#### DNA Methylation

**Figure 1:**
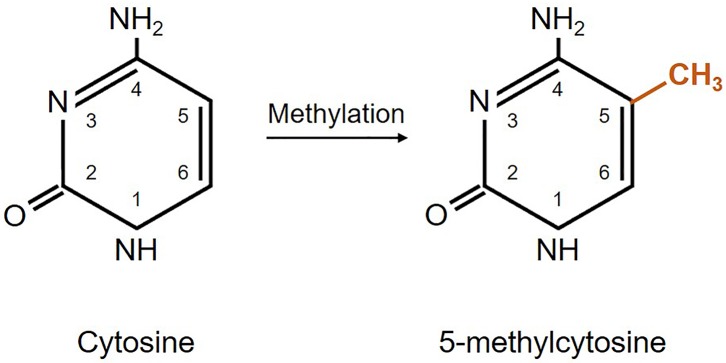
Schematic representation of DNA methylation. Cytosine methylation is mediated by a family of DNA methyltransferase enzymes DNMT1, DNMT3A, and DNMT3B (65, 80, 81).

From the four DNA bases, only cysteine and adenine can be methylated. Yet, DNA methylation usually refers to the covalent transfer of methyl groups to the C-5 position of the cytosine ring to create 5-methylcytosine as shown in Figure 1. In mammals, DNA methylation occurs on any cytosine of the genome; however, in the majority of the cases it occurs in a 5′-CpG-3′ dinucleotide context of somatic cells, and up to 25% of methylation occurs in non-CpG context of embryonic cells (82). Typically, CpG islands belong to or are near promoter regions of genes where transcription starts (74). Adenine methylation is observed in bacterial, plant, and lately also in mammalian DNA, but is not that much explored and its role is largely unknown (83–85). Methylation is needed for silencing transposable elements and genes on the inactive X-chromosome, as well as maintaining genome stability (86). Besides, DNA methylation plays an important role in regulation of gene expression that has an impact on the clinical outcome of glioblastoma patients (65, 87, 88).

#### DNA Methylation in Glioblastoma

Cancers in general are characterized by global hypomethylation which is associated with gene expression, activation, and chromosomal rearrangements of oncogenes which leads to genomic instability, oncogene activation, and tumor progression, as well as locus-specific hypermethylation which results in heritable transcriptional silencing of tumor suppressor genes (82, 89). Global hypomethylation occurs in 80% of glioblastomas (90). DNA methylation that occurs in CpG islands in gene promoter regions inversely correlates with gene transcription. In glioblastomas, besides at genetic, intratumor heterogeneity is present also at DNA methylation level. Wenger et al. analyzed multiple spatially separated tumor specimens from 12 glioblastoma patients (38 samples total) and observed existence of different methylation subclasses intertumorally—three samples presented with combined existence of mesenchymal and receptor tyrosine kinase (RTK) II subclasses, while two contained both RTKI and RTKII at once (91). Using clustering of *IDH*-mutant gliomas, Ceccarelli et al. observed existence of three major glioma groups: Codel, *IDH*-mutant 1p/19q codeleted low grade gliomas; glioma CpG island methylator phenotype (G-CIMP)-low, *IDH*-mutant non-1p/19q codeleted low and high grade gliomas with low genome-wide DNA methylation; and G-CIMP-high, *IDH*-mutant non-1p/19q codeleted low and high grade gliomas with higher level of genome-wide DNA methylation (92). Among these, the G-CIMP low group was found to have worst prognosis, while G-CIMP and Codel presented with similar favorable prognoses. The authors also analyzed *IDH*-wild type gliomas and observed existence of three DNA methylation groups: classic-like, mesenchymal-like, and *IDH*-wildtype low and high grade gliomas. An interesting observation was that only patients with low grade gliomas from the *IDH*-wildtype group experienced significantly longer survival. In a different study, Pangeni et al. performed methylation array profiling on a panel of 23 patient-derived glioblastoma stem cell lines and data for TCGA patients with *IDH*-wildtype glioblastomas (89). Different glioblastoma subtypes were included in the analysis. The authors observed similar bi-modal distribution of CpG methylation in glioblastoma stem cells and glioblastoma tumors. They also found more mesenchymal-hypermethylated than hypomethylated genes in both glioblastoma stem cells and glioblastoma tumors. They observed that high expression levels of proneural-methylated genes CASP8 and FADD-like apoptosis regulator (*CFLAR*) and Sp100 nuclear antigen (*SP100*), and low expression levels of the mesenchymal-methylated genes transmembrane and coiled-coil domain family 1 (*TMCC1*), Rho guanine nucleotide exchange factor 7 (*ARHGEF7*), Notch homolog 1, translocation-associated (*NOTCH1*), midnolin (*MIDN*), potassium voltage-gated channel subfamily Q member 2 (*KCNQ2*), ataxin 10 (*ATXN10*), ubiquitin specific peptidase 54 (*USP54*), and TUB bipartite transcription factor (*TUB*) correlate to poorer patient prognosis (89).

In glioblastoma, DNA methylation is closely correlated to response to temozolomide treatment. So far, methylation of the O^6^-methylguanine-DNA methyltransferase (MGMT) is the only predictive biomarker for patient response to first-line temozolomide chemotherapy (93, 94). MGMT is a DNA repair protein that reverses the damage done by alkylating agents such as temozolomide. Temozolomide adds methyl groups at N^7^ and O^6^ sites on guanines and O^3^ sites of adenines in genomic DNA and this is why it is cytotoxic to cells (14). *MGMT*-promoter methylation causes gene silencing, therefore less protein is expressed which leads to sensitivity to chemotherapy with alkylating agents (95). In a study by Smrdel et al. the authors compared overall survival and time to progression in patients with and without methylated MGMT (96). Their results show longer overall survival (43 vs. 16 months) and time to progression (36 vs. 11 months) in patients with methylated MGMT compared to patients without methylated MGMT, respectively. The authors also observed MGMT methylation in 36 out of 38 (95%) patients who present with long survival (more than 30 months after diagnosis) and in only 12 out of 33 (36%) patients in the control group (short term survival patients i.e., <30 months after diagnosis). In general, patients with methylated *MGMT* promoter respond better to temozolomide therapy and present with longer survival (3, 63, 97, 98). These findings are in concordance with another study where Felsberg et al. took in consideration 67 adult patients diagnosed with glioblastoma (99). The authors conclude that *MGMT* promoter hypermethylation is associated with longer time to progression after initiation of chemotherapy (245 vs. 100 days) and longer overall survival (692 vs. 474 days).

On the other hand, one study reports identification of a new histone deacetylase inhibitor, 7-ureido-N-hydroxyheptanamide derivative—CKD5, that shows strong anticancer effect in glioblastoma *in vitro* and *in vivo* (100). The advantages of this inhibitor are its water solubility (>1,000 mg/mL), negative result on Ames test which indicated that CKD5 is not genotoxic and does not introduce DNA mutations, and it showed no signs of inducing cardiac toxicity in pre-clinical trials. However, for glioblastoma treatment delivery methods have to be developed as CKD5 cannot penetrate the blood brain barrier. The use of H3K4 and H3K9 me1/2 demethylase KDM1 and H3K4 me2/3 demethylase KDM5A as potential therapeutic targets was also tested (76). The authors of the study show that inhibiting KDM1 and KDM5A enzyme activity presents with strong antitumor affect in wild-type and temozolomide-resistant glioblastoma cells. Romani et al. used a multi-KDM inhibitor, JIB 04, that has strong anti-clonogenic activity in wild-type and temozolomide-resistant glioblastoma cells after only 4 h incubations at low JIB 04 concentrations (0.5 and 1 μM). The authors also tested different drug combinations and showed synergistic effect of JIB 04 and GSK J4 (selective inhibitor of KDM6A/B) on cell proliferation and reduction of the clonogenic potential of temozolomide-resistant glioblastoma cells. These studies show that with targeting epigenetic changes non-traditional treatment methods for glioblastoma patients whose tumors are resistant to the temozolomide chemotherapy can be developed. Knowing the aggressiveness of the tumor and its poor response to current available treatment options, these findings give new hope for glioblastoma patients.

#### Clinical Trials

Since the discovery of the altered epigenetic profiles of cancers, epigenetics is getting more and more attention in the research community. Histone deacetylases, the enzymes that remove the acetyl group from histones which is initially associated with gene repression, are often overexpressed in cancer (100). Due to their reversible nature and role in gene expression, epigenetic changes, especially changes in histone acetylation, are also considered as possible therapeutic targets (86) which can be seen from the growing number of clinical trials based on the use of different enzyme inhibitors. Data was obtained from the database of publicly and privately funded clinical studies worldwide, https://clinicaltrials.gov, using the following keywords: “glioblastoma,” “Vorinostat,” “Romidepsin,” “Belinostat,” “Panobinostat,” “LHB589,” “Valproic acid,” “Olaparib,” “Veliparib,” “ABT-888,” “Iniparib,” “BSI-201,” and “CBL0137.” The search conducted on the 8th of August 2019 gave the results presented in Table 1. Still, an obstacle in these clinical trials are our current lack of knowledge about the mechanism of HDAC inhibitors and how they affect cellular signaling pathways; moreover, methods for improved penetration of the HDAC inhibitors into the brain and across the blood brain barrier are still to be found (77).

**Table 1:** List of selected glioblastoma clinical trials using drugs targeted against enzymes involved in epigenetic modifications.

**Drug**	**Role**	**Clinical trial number**	**Clinical trial phase**	**Selected references**
Vorinostat	HDAC inhibitor	NCT00762255 NCT01266031 NCT01110876 NCT03426891 NCT01342757 NCT00555399 NCT00731731 NCT00641706 NCT00238303 NCT00939991 NCT01738646 NCT01189266 NCT00268385 NCT01378481	I (completed) I (completed) I (terminated) I (recruiting) N/A I/II I/II II (completed) II (completed) I/II II (completed) I/II I I (terminated)	(101–107)
Romidepsin	HDAC inhibitor	NCT00085540	I/II (completed)	(108)
Belinostat	HDAC inhibitor	NCT02137759	II	(109)
Panobinostat (LBH589)	HDAC inhibitor	NCT01115036 NCT00848523	II (withdrawn) II (terminated)	(110)
Valproic acid	HDAC inhibitor	NCT02648633 NCT02758366 NCT01817751 NCT01861990 NCT03243461 NCT00302159	I (terminated) II II I (withdrawn) III II (completed)	(111–117)
Olaparib	PARP inhibitor	NCT02974621 NCT01390571 NCT03212274	II I (completed) II (recruiting)	(118, 119)
Veliparib (ABT-888)	PARP inhibitor	NCT02152982 NCT00770471 NCT01026493 NCT03581292 NCT01514201	II/III I (completed) I/II (completed) II (recruiting) I/II (completed)	(120, 121)
Iniparib (BSI-201)	PARP inhibitor	NCT00687765	I (completed)	(122)
CBL0137	Histone chaperone FACT inhibitor	NCT01905228	I (recruiting)	(123–125)

*HDAC, histone deacetylase; PARP, poly(ADP-ribose) polymerase; FACT, facilitates chromatin transcription; N/A, not applicable*.

### Novel Next Generation Sequencing-Based Diagnostic Approaches

Although the work of Verhaak et al. changed the way we diagnose glioblastoma and increased our understanding of the disease (50), there are still a number of patients whose tumors cannot be classified according to the currently defined subtypes (13). Anyway, understanding the molecular background of disease requires availability of sets of reference samples from healthy donors (126) for comparison.

#### Molecular Re-classification of Glioblastomas

Using next generation sequencing data available from TCGA, Bolouri et al. report clustering of glioblastomas based on their methylation profiles (127). The authors of the study used genome wide and methylation data from a merged cohort of glioblastomas and lower grade gliomas and obtained three big glioma clusters: (1) oligodendrogliomas, (2) astrocytomas and oligoastrocytomas, and (3) mostly glioblastomas with a few astrocytomas and oligoastrocytomas (127).

More recently, Capper et al. aided in the classification of glioblastomas that do not belong to the known entities i.e., WHO subclasses (128). The authors established a system for classification of brain tumors based on their epigenetic profiles. They generated genome-wide DNA methylation profiles of 76 histopathological entities and seven entity variants that occur in the central nervous system. Unsupervised clustering within each entity and across histologically similar tumor entities led to obtaining 82 tumor classes with different DNA methylation profiles. Of these, 29 classes were equivalent to single WHO entities, 29 classes were subclasses of WHO entities, in eight classes WHO entities could not be recapitulated, in 11 classes were not identical to WHO entities, while the remaining five classes presented with methylation profiles that are not described by the WHO. To test clinical implementation of this system, Capper et al. analyzed 1,104 samples or 64 different histopathological entities of adult and pediatric brain tumors. The majority of the samples (88%) matched an established DNA methylation profile. From these, in 76% of the cases pathological and methylation profiling were concordant. In the remaining 12% of the cases, methylation and pathological diagnoses were not concordant; samples were molecularly re-evaluated and diagnosis was changed as it was suggested by the methylation subclassifier; diagnoses were changed into both lower (30%) and higher (41%) WHO grades. The study demonstrates that variability within and among tumors can be detected with deeper molecular analysis which can lead to more precise diagnosis and better treatment.

Cancer methylome is a combination of DNA methylation changes and characteristics of the cells of origin; in heterogeneous metastatic tumors this can aid in defining the primary cancer site (128). Although adding molecular characteristics into the histological diagnosis of glioblastoma is beneficial for precise diagnosis, single-gene tests based on DNA methylation analysis like *MGMT* methylation status, fluorescence *in situ* hybridization [1p/19q codeletion, *EGFR*, proto-oncogene C-Myc (*MYC*), class E basic helix-loop-helix protein 37 (*MYCN*), platelet derived growth factor receptor alpha (*PDGFRA*) and 19q13.42), or immunohistochemistry (catenin beta-1 (CTNNB1) and Lin-28 homolog A (LIN28A)], have proven difficult to be standardized (128). The studies from Bolouri et al. and Capper et al. only illustrate the importance of implementation of methylation profiling in glioblastoma diagnosis. Due to tumor heterogeneity it is important for all variables to be taken in consideration for fully informed decision about patient's therapeutic course to be made.

#### Nanopore Sequencing for Same-Day Patient Diagnosis

Further development of next generation sequencing techniques may lead to same-day patient diagnosis with nanopore-based systems. Similar to a small 100 mV electrical current passing through a nanopore placed in a membrane separating two chambers with aqueous electrolytes that can be measured with standard electrophysiological techniques, nucleobases of electrophoretically driven ssDNA or RNA molecules would modulate the current when passing through a suitable nanopore (129). The ideal nanopore sequencer is characterized by inexpensive sample preparation complemented with disposable chip with integrated microfluidics and probes, and a portable benchtop instrument for processing of long reads (>10,000–50,000 nt). Using a nanopore sequencer, Jain et al. reported sequencing and assembly of a reference human genome from ultra-long reads up to 882 kb in length with 5x coverage (130). The advantage of this benchtop method over next generation sequencing is its simplicity, speed, size, and reduced cost—nanopore can provide sequencing results faster and in resource-restricted areas (44). One big concern however, is the amount of starting material; namely, ~700 μg of human diploid genetic material will be needed to provide adequate throughput for the nanopore array, which, in theory, can be obtained with routine extraction procedures (129). Still, in the time when single-cell sequencing research is escalating (52, 55, 131) this can be seen as a pitfall.

Meanwhile, nanopore sequencing is already being tested for implementation in cancer diagnostics. In their retrospective proof-of-principle study, Euskirchen et al. examined the utility of nanopore sequencing (Oxford Nanopore Technologies, Oxford, UK) for multimodal molecular diagnosis using previously characterized brain tumors (132). Using deep amplicon sequencing, the authors were able to identify single nucleotide variants in *IDH1, IDH2, TP53, H3F3A*, and *TERT* promoter, and diagnostically relevant alterations like 1p/19q codeletion within minutes of sequencing. Moreover, they obtained 0.1X genome coverage within 6 h where copy number and epigenetic profiles matched the ones from microarray data. Because it can detect base modifications such as 5-methylation of cytosines, nanopore sequencing is also suitable for methylation analysis without the need of bisulfite conversion of the DNA. This will increase consistency during nucleic acid processing as well as significantly reduce the time needed for sample preparation. In the study, the authors observed good correlation between the methylation events in CpG sites obtained with nanopore sequencing and the corresponding microarray data.

The study by Euskirchen et al. shows the potential that nanopore sequencing offers for timely cancer diagnosis. In addition, due to small size and inexpensive technology, this method of histomolecular disease classification can be used worldwide even in regions with restricted clinical resources. However, further optimization like for use with highly fragmented nucleic acids originating from improperly stored or formalin-fixed paraffin-embedded tissue samples is still needed. Furthermore, as the method is still in developmental stages frequent improvements of the chemistry and software challenge its clinical implementation at this time.

### Artificial Intelligence in Biomedical Sciences

In the light of new technological developments, the use of artificial intelligence (AI) in biomedical research will bring glioblastoma diagnostics on an advanced level. Machine learning is an application of artificial intelligence that allows for computers to work on tasks, learn from the data and improve their performance based on the gained experience (133, 134). It is a combination of mathematics and computer science that is based on building statistical models from large datasets i.e., billions or trillions data points (133). Classical statistical models describe the relationship between covariates (e.g., clinical factors) and a single dependent variable (e.g., outcome) obtained from a sample population and projected to a larger population. For instance, statistical models are suitable for deciding on treatment strategies based on survival, while machine learning models seek to predict the outcome using clinical factors as input features (135). Machine learning is defined as “the study of algorithmically built mathematical models that have been fitted for the training dataset to make predictions for the similarly obtained and structured validation dataset” (136). Extensive use of machine learning in biomedical fields, either for diagnostic, or therapeutic purposes, is conditioned by the availability of large data sets and appropriate IT infrastructure. Large datasets containing genetic information are generated by sequencing the human genome—a method that became routine with the wide implementation of next generation sequencing in research.

#### Machine Learning for Disease Diagnosis and Therapy

In cancer diagnostics, microscopic examination of patient samples is crucial for determining cancer staging. However, microscopy is based on the image interpretation of an expert individual which can also be subjective; lately quantitative examination of microscopy samples is required; and lastly, the availability of such expert individuals can be limited (137). AI can help in automated image analysis for pathological purposes with improved diagnostic accuracy, quantification, and efficiency. One such example is the augmented reality microscope—optical light microscope that enables real-time integration of AI (137).

Generation of large amount of data that contains information about human genetics allows for the development of machine learning techniques whose algorithms are either supervised or unsupervised clustering type. In supervised learning algorithms learn from labeled data, while in unsupervised learning the algorithms try to understand relationships from unlabelled data (134).

Machine learning for therapeutic purposes will be additionally enriched by the implementation of *in silico* drug discovery and design systems. One such example is the DrugBank database that contains quantitative, analytic and molecular information about drugs, and drug targets (138). DrugBank is organized into four major groups: (1). FDA approved small molecule drugs (>700 entries), (2). FDA approved bio-tech (protein/peptide) drugs (>100 entries), (3). Nutraceuticals or micronutrients such as vitamins and metabolites (>60 entries), and (4). Experimental drugs such as unapproved, de-listed and illicit drugs, enzyme inhibitors, and potential toxins (3,200 entries). Machine learning for drug discovery will offer a cost-effective and timely alternative to current experimental procedures (139). Another perspective is applying machine learning technology for predicting clinical efficiency of drugs and individualized treatment methods (140). This method which is named “drug scoring” or “personalized (individual) medicine” will take into account features that describe activation of cell signaling and metabolic pathways which will distinguish patients who will respond to the treatment from those who will not benefit from it. There are two principles for drug scoring: *a priori*—evaluating the ability of a drug to restore normal status or stop a physiological process that is considered pathogenic; and *a posteriori*—resulting from a machine learning process on a training dataset containing information from patients treated with the drug in question (140). The authors have developed a method for translating drug efficiency results obtained using cell lines to predict clinical effect on humans. The use of this method can potentially reduce the costs of drug screening.

Even though machine learning shows potential to improve disease diagnosis and therapy, it still possesses a few limitations such as separating causation from correlation, removing biased data, and regulating predictive analytics (141). For machine learning to be safely used in disease diagnosis and/or treatment, the data which is taken into consideration has to be thoroughly examined to ensure it is appropriate for the specific problem. For correct identification and analysis, data must be equally collected and annotated, and it must be representative even for minorities in heterogeneous populations. In addition, self-implemented risk factors like smoking should be taken in consideration but should not be a limiting factor when deciding on future healthcare measures. An ethical issue in deciding treatment with machine learning can be existence of a permanent health condition, or chronic infection, like an individual being HIV positive. Another possible problem is bias toward populations that provide the most data, and in some societies, toward those that are able to afford medical procedures. As machine learning algorithms are trained on retrospective data it is possible for human subjectivity to influence the results; however, this can be improved by introducing new raw data (135). Before applying machine learning into clinical care, researchers must also consider protecting the privacy of the patients, ensure protection of data and patient information, and allow for equal treatment of all affected parties (141). At last, validation and independent testing of the machine learning algorithms must be performed in order to exclude mistakes due to technical differences. With all the advantages that it offers, machine learning in biomedicine is still at the beginning of its development and requires a multidisciplinary team to answer questions about ethical, legal, moral, and technological issues before it can objectively aid in better patient care.

#### Deep Learning

Machine learning works only with structured data which means reduction of amount of data in the raw format, significant time input from a medical professional to structure the data and introduction of human subjectivity (135). On the other hand, deep learning can use a wide range of different parameters which can be optimized by training on labeled data for prediction. While machine learning has already been used to determine gene expression patterns relevant for glioblastoma patient survival (50, 142), the use of deep learning for prognostic gene discovery it at its beginnings (143). The advantage of deep learning is that it can model a large number of differentially expressed genes. Using TCGA data as input, Wong et al. used deep learning to model the relationship between genes and their corresponding proteins on survival prognosis (143). Their model identified different genes associated with glioblastoma survival, glioblastoma cancer cell migration, or glioblastoma stem cells. In a different study, Young et al. used deep learning to classify glioblastomas into six subtypes which corresponded with significant differences in patient survival (144). These findings are in concordance with those from Brennan et al. who used DNA methylation data to classify glioblastomas into six subtypes: mesenchymal, proneural, neural and classical, as identified by Verhaak et al., supplemented with G-C island methylation phenotype (G-CIMP) and non-G-CIMP subtypes within the proneural subtype (145).

**Figure 2:**
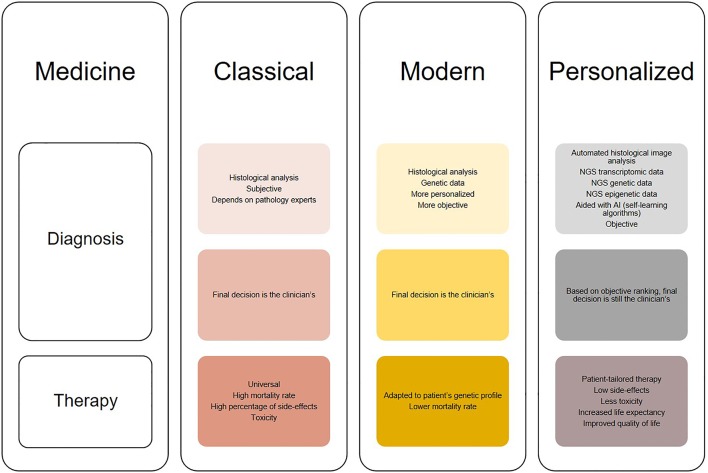
Illustration of the changes in diagnosis and treatment of glioblastomas in different medical approaches (classical, modern, and personalized). Classical medicine relied on histological analysis of tissues, which is merely subjective, while therapy is universal for the patients which does not show much clinical success. On the other hand, modern medicine takes in consideration both histologic and genetic components which leads to greater diagnostic accuracy [examples: “glioblastoma, IDH-mutant” and “oligodendroglioma, IDH-mutant and 1p/19q-codeleted” (13)], while therapy is modified to suit tumor genetic profile. The trend is moving toward personalized medicine, where diagnosis will be thorough and objective aided by automated histological image analysis, next generation sequencing (NGS) and artificial intelligence (AI) algorithms, and therapy will be adapted not only to the genetic but also transcriptomic and epigenetic patient profile. This will result in increased overall survival and better quality of life.

**Figure 3:**
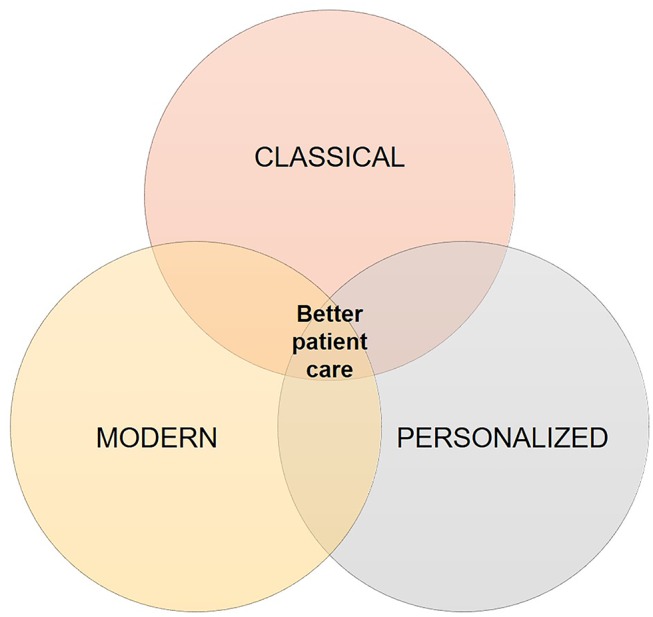
Glioblastoma diagnosis. This figure illustrates what all healthcare systems have in common and aim for—which is better patient care. Starting from the classical, to the modern, where we are now, and going toward personalized medicine the goal is still improving patient quality of life i.e., both disease diagnosis and patient treatment.

Although still at its infancy, the use of deep learning may open up new possibilities for glioblastoma diagnosis. Due to its ability to analyse large amount of data, deep learning can aid identifying features with biological significance which are currently unknown or too complex to be understood. Moreover, novel applications of deep learning may reveal hidden structures of cellular pathways and disease mechanisms. Glioblastoma diagnosis has significantly changed through the years as it can be seen in Figure 2. It started from histological analysis (classical) through addition of molecular and genetic characteristics of the individuals (modern) and is moving toward implementation of self-learning algorithms (personalized) which will eventually lead to the next presently unknown level. However, all these “medicines” have a common goal that is longer life expectancy and greater quality of life or better patient care (Figure 3). This transition from the classical through modern to personalized medicine was greatly aided by the massive use of NGS methods and is able to further develop also because of them.

## Conclusions

With the use of NGS researchers generate large amount of data about transcriptomic, genomic, and epigenomic characteristics of humans. However, so far, only a small fraction has been proven to have clinical implementation. Understanding the rest of the “unlocked” data will only be possible with the development of more powerful analytic objective systems. The potential that NGS holds for glioblastoma research and clinical implementation is massive. Intelligent and careful use of NGS data can change the way we diagnose and treat glioblastomas. Studying epigenetic modifications in glioblastoma offers potential for identification of clinical biomarkers either for patient diagnosis or discovering drug targets. Rapid development of different methodologies for analysis of big data may lead to the development of individual diagnosis and patient-tailored treatment. It is expected for *in silico* diagnosis to be comparable and consistent, less variable, objective, and without human error. However, machine learning and deep learning algorithms have still a lot to learn before this diagnostic model can be implemented in everyday clinical practice. At last, these models need to be trained to understand biological systems so they can have an “insight” into the disease biology. This way, machine learning and deep learning models can adapt their findings in concordance to the nature of the analyzed disease, and simultaneously learn and change as the disease evolves.

## Author Contributions

IJ performed literature search, collected data and information, prepared figures and table, and wrote the manuscript.

## Conflict of Interest

The author declares that the research was conducted in the absence of any commercial or financial relationships that could be construed as a potential conflict of interest.
